# Brown adipose tissue: can it keep us slim? A discussion of the evidence for and against the existence of diet-induced thermogenesis in mice and men

**DOI:** 10.1098/rstb.2022.0220

**Published:** 2023-10-23

**Authors:** Jan Nedergaard, Gabriella von Essen, Barbara Cannon

**Affiliations:** Department of Molecular Biosciences, The Wenner-Gren Institute, Stockholm University, SE-106 91 Stockholm, Sweden

**Keywords:** diet-induced thermogenesis, UCP1, brown adipose tissue

## Abstract

The issue under discussion here is whether a decrease in the degree of UCP1 activity (and brown adipose tissue activity in general) could be a cause of obesity in humans. This possibility principally requires the existence of the phenomenon of diet-induced thermogenesis. Obesity could be a consequence of a reduced functionality of diet-induced thermogenesis. Experiments in mice indicate that diet-induced thermogenesis exists and is dependent on the presence of UCP1 and thus of brown adipose tissue activity. Accordingly, many (but not all) experiments indicate that in the absence of UCP1, mice become obese. Whether similar mechanisms exist in humans is still unknown. A series of studies have indicated a correlation between obesity and low brown adipose tissue activity, but it may be so that the obesity itself may influence the estimates of brown adipose tissue activity (generally glucose uptake), partly explaining the relationship. Estimates of brown adipose tissue catabolizing activity would seem to indicate that it may possess a capacity sufficient to help maintain body weight, and obesity would thus be aggravated in its absence.

This article is part of a discussion meeting issue ‘Causes of obesity: theories, conjectures and evidence (Part II)’.

## 1. Introduction

It is now some 60 years since the heat-producing role of brown adipose tissue was established: the heat from brown adipose tissue keeps mammals warm and helps them arouse from hibernation [1–3]. It took another 15 years to fully formulate that heat production—in brown adipose tissue or, of course, anywhere in the body—necessarily uses food energy; this means that activated brown adipose tissue can combust some of the energy in the food, or even some energy already stored in the organism. If this occurs under conditions where the heat is not needed for thermoregulatory purposes, we have a situation of ‘diet-induced thermogenesis’. This was principally what was formulated by Rothwell and Stock in 1979 [4], including that this thermogenesis probably originated in brown adipose tissue. Thus, diet-induced thermogenesis would protect against obesity.

The thought that obesity may be caused by a lower activity of brown adipose tissue, i.e. a lower diet-induced thermogenesis, has since then attracted much attention, particularly as reflected by the idea that hyperactivation of brown adipose tissue, or enhanced diet-induced thermogenesis, could actively lower the degree of obesity. The realization that active brown adipose tissue is found in adult humans ([5] and many later papers) has vastly amplified the interest in the phenomenon: does brown adipose tissue keep us slim?

We examine below evidence for the existence or not of (brown-fat-derived) diet-induced thermogenesis, first in experimental systems (mice) and then in humans (to the extent it exists).

## 2. Why would diet-induced thermogenesis exist?

The existence of a mechanism for combusting food energy without saving the released energy in the form of ATP—but releasing it just as heat—is obviously contrary to general ideas that evolution would always seek to maximize energetic efficiency. It is thus only evolutionarily understandable if other benefits would be associated with such a mechanism. Here, the generally accepted hypothesis for the existence of the phenomenon of diet-induced thermogenesis is that this process would make it possible for animals to survive on a diet low in e.g. proteins. Animals would overeat the diet so that sufficient amounts of proteins were obtained [6,7]. However, the extra food energy thus consumed and potentially transferred to extra lipid stores could be a burden for survival. Thus, if that extra food energy were to be combusted, the problem disappears, and the animals would stay slim despite overeating energetically. Enthralling as this theory may seem, it has not been experimentally easy to prove. Although brown adipose tissue recruitment and some overeating, as well as enhanced energy expenditure, may indeed be induced by a low protein diet, the overeating does not experimentally suffice to compensate for the low protein content in the diet and to allow for full growth rates (e.g. [8]; our unpublished observation, 2023).

Additionally, this model—that may be said to encompass some kind of protein sensing—obviously does not easily extrapolate to the conditions presently associated with diet-induced thermogenesis, i.e. an increased heat production apparently caused by overeating a tasty diet such as the routinely used ‘high-fat diets’. Such diets contain sufficient protein so that any diet-induced thermogenesis observed cannot be a response to low protein—but it could be a response to increased adiposity as such.

## 3. Does diet-induced thermogenesis exist?

The possibility that brown adipose tissue (when active) keeps us slim thus depends fully on the actual existence of brown-fat-mediated diet-induced thermogenesis. A criterion for its existence would be that it is thermogenesis induced by a diet that inspires overeating. To demonstrate the existence of diet-induced thermogenesis, it is, however, not sufficient to demonstrate a higher rate of thermogenesis in animals that overeat. This is because the total thermogenic response to eating—that we can call ‘diet-induced thermogenesis’—includes what is referred to as ‘obligatory’ diet-induced thermogenesis [9]. This is the extra heat inevitably produced by the organism in the processes of digesting the food and transferring the energy into lipids, etc. Rather, the diet-induced thermogenesis discussed here should be both *facultative* and *adaptive*; this means that it should only occur when it is ‘needed’ (food is acutely obtained, i.e. it is facultative), and it should only occur when the organism has been exposed to excess energy intake for a prolonged time (adaptive). It is in this context that we use the term below. Finally, in the present formulation, it should be associated with recruitment of brown adipose tissue. These criteria are thus parallel to those established for classical non-shivering thermogenesis: facultative (only in the acute cold), adaptive (only recruited after prolonged time in the cold) and brown-fat-derived.

## 4. Diet-induced thermogenesis may exist and is dependent on UCP1/brown adipose tissue

**Figure 1.**
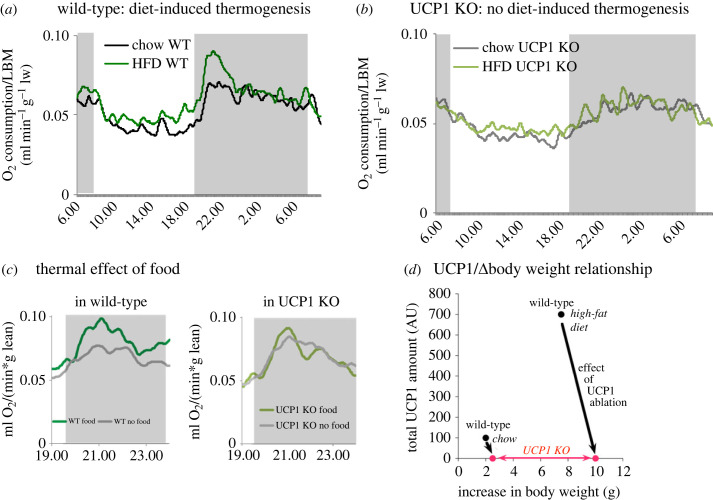
Diet-induced thermogenesis in mice, demonstrating adaptiveness, facultativeness and UCP1 dependence. (*a*) Wild-type mice (C57Bl/6), living at thermoneutrality, were fed a normal chow diet or a high-fat diet (HFD) for four weeks. They were then placed in an indirect calorimeter at thermoneutrality and their rate of oxygen consumption followed during night (grey shadow) and day. As seen, both chow- and high-fat-fed mice display a low rate of oxygen consumption during the rest (day) period, but shortly before the night period starts, the mice become active and their oxygen consumption rate increases. However, the increase is much higher in the mice that have been fed the high-fat diet (i.e. it is adaptive). The results in *a*–*c* are given per gram lean body mass. (*b*) UCP1 KO mice examined in parallel to the wild-type in *a*. The basic pattern is the same but the higher increase in oxygen consumption rate seen in wild-type is not seen in UCP1 KO mice; thus, no adaptive thermogenesis is evident in these mice. (*c*) Response to feeding. Mice, placed in an indirect calorimeter, that had been fed a high-fat diet for four weeks, had either normal access to food (green line) or were deprived of food just before night time (grey line). Left: wild-type mice: feeding induces a higher rate of oxygen consumption than lack of food, thus it is facultative. Right: no such facultative thermogenesis is seen in UCP1 KO mice. (*d*) The relationship between body weight increase (obesity) and total amount of UCP1 in the interscapular brown adipose tissue depot. When the wild-type mice were exposed to a high-fat diet, their total amount of UCP1 was increased and so was their body weight (black dots). When the UCP1 KO mice were exposed to high-fat diet, there was evidently no increase in UCP1 amount, but their body weight increased more than it did in the wild-type mice (red dots). The black arrows thus indicate the ‘effect’ of the lack of UCP1 on the body weight. All curves redrawn (with broader time smoothing) from von Essen *et al.* [13], where further methodological detail is also described.

Although the existence of diet-induced thermogenesis has been and is doubted [10–12], it would seem that it can be demonstrated to occur. We have found [13] that in mice chronically exposed to a—tasty—high-fat diet, the thermogenic response to eating is higher than in mice fed a chow diet (figure 1*a*). That this response is larger than that in mice chronically exposed to a chow diet is not explainable by an enhanced *obligatory* diet-induced thermogenesis. This is because, at this time point, the mice on a high-fat diet do not eat more than those on a chow diet, and although the composition of the diet is different, the difference is a change from carbohydrate to fat in the diet, i.e. to a nutrient with even lower obligatory thermogenesis. The increased thermogenic response to the meal has not, however, fully counteracted the accumulation of extra energy in the body: the high-fat-fed mice still become more obese than the chow-fed mice (figure 1*d*, body weight changes on *x*-axis). The higher thermogenesis is only seen in wild-type mice; in the UCP1 knockout (KO) mice, there is no adaptive thermogenesis (figure 1*b*). Further, the thermogenesis is only seen as a direct response to feeding (figure 1*c,* left), and this feeding response is dependent on the presence of UCP1 and is thus not seen in UCP1 KO mice (figure 1*c,* right).

## 5. Obesity correlates positively with brown adipose tissue recruitment and increased total UCP1 amounts

Importantly, the increased thermogenic response to a meal is correlated with a higher amount of UCP1 in the wild-type mouse (figure 1*d*). Thus, in this homeostatic system, a high-fat diet initially leads to increased food intake, and the mice become more obese, but they combust with time a larger fraction of the food, probably in brown adipose tissue, since they have more UCP1. That the system is effective is seen (figure 1*d*) in that, given access to the same food, the mice without UCP1 become even more obese. That the system is only *partly* efficient is seen from the fact that even the UCP1-containing wild-type mice still become fatter when exposed to the high-fat diet than when exposed to the chow diet (figure 1*d*).

## 6. Two apparently contradictory statements

At first sight, it may be somewhat confusing that two, apparently contradictory, statements coexist: that obesity as such *drives* activation of brown adipose tissue recruitment and increases in UCP1 amounts, i.e. leading to diet-induced thermogenesis (as seen above), and that obesity may rather be *caused by* lowered brown adipose tissue activity. These statements are, however, not as contradictory as they seem: the increase in UCP1 amount and diet-induced thermogenic capacity is what is supposed to occur in the ‘healthy’ animal under homeostatic control. If this does not happen, or if UCP1 and brown adipose tissue in any other way are less effective, obesity would develop.

## 7. Does the absence of UCP1 lead to obesity?

**Figure 2.**
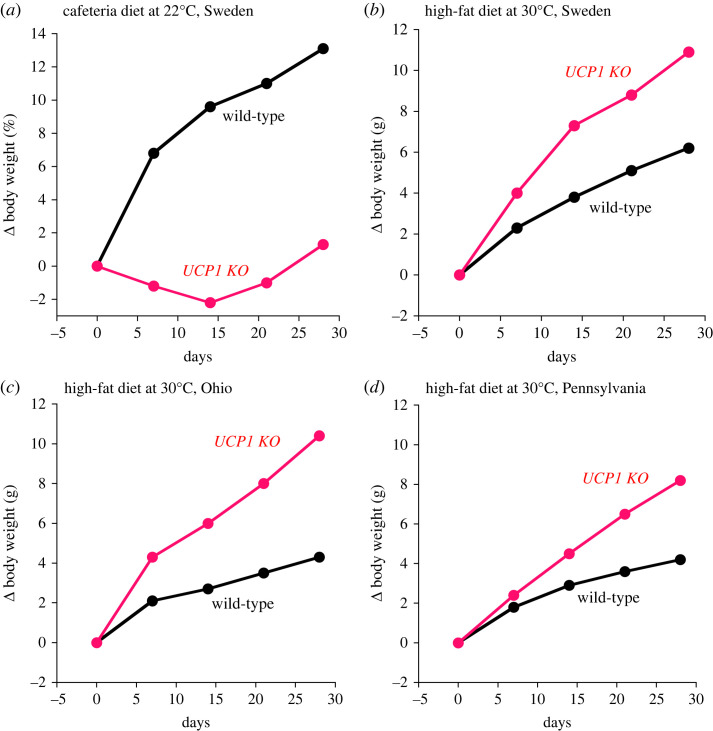
Reproducibility of obesity-inducing effect of UCP1 ablation in different laboratories. (*a*) Wild-type (black) and UCP1 KO mice (red) on an FVB/N background kept at normal housing temperatures (≈22°C) were exposed to a cafeteria diet and the body weight followed for four weeks. Our unpublished observation (performed 2005). (*b*) Wild-type (black) and UCP1 KO (red) mice of the C57Bl/6 strain kept at thermoneutrality (≈30°C) were exposed to a high-fat diet and the body weight followed for four weeks. Data redrawn from Feldmann *et al.* [16]. (*c*) Similar to the experiment in *b* but performed in Ohio. Data redrawn from Rowland *et al.* [17]. (*d*) Similar to the experiment in *b* but performed in Pennsylvania. Data courtesy of John G. Geisler.

Following the concepts above, the prediction would be that the absence of UCP1 in itself should lead to overt obesity, similarly to the obesity-inducing effect of the absence of leptin in the *ob/ob* mouse. However, in the initial studies of the UCP1 KO mice, obesity was not observed. Already in the first report on the UCP1 KO mice [14], it was pointed out that there was no evidence for spontaneous obesity, and Liu *et al*. [15] in a high-fat diet study not only failed to see augmented obesity but even observed protection from obesity. We observed similar results (figure 2*a*) such that both when UCP1 KO mice, in this case on an FVB/N background, were fed a chow diet (not shown here) or a high-fat cafeteria diet, the KO mice were protected against obesity, as was also observed on a C57Bl/6 background [15,18]. There is still no explanation for this obesity-protecting effect of UCP1 ablation in C57Bl/6 mice under normal animal house conditions; there is no evidence for marked changes in food intake or ongoing thermogenesis, but small changes over prolonged periods may perhaps be responsible [18]. Remarkably, in mice lacking both UCP1 and FGF21, the mice again become obese on a high-fat diet [18]; the reason for this is also presently unknown.

## 8. Why is there no obesity-promoting effect of UCP1 knockout at normal housing temperatures?

Considering the situation from a thermoregulatory perspective, it may—in retrospect—be evident that we should not expect to observe any effect of UCP1 ablation under ‘normal housing’ conditions. This is because at normal animal house conditions (≈20°C), the mice experience significant cold, and they must increase their metabolism by 50–100% (as compared to mice living at thermoneutrality) to compensate for the heat loss (e.g. [19]). This clearly means that any heat production derived from diet-induced thermogenesis will be used in the struggle to keep up the body temperature: the mice have to produce extra heat or they will become hypothermic and die. Thus, any heat from any diet-induced thermogenesis will merely be experienced as extra heat—but if this is absent, it has to be substituted for with some other ‘heat’. Thus, UCP1 KO mice have to produce the same amount of heat as have wild-type mice to survive under normal housing conditions. How this is accomplished is presently not really clarified. Even under normal housing conditions, UCP1 KO do not show overt shivering (as they do when exposed to the much greater ‘standard’ cold (=4°C) [20]. As the UCP1 KO mice are devoid of adrenergically induced non-shivering thermogenesis [21], the heat must be produced in other ways, possibly through increased muscular tone [22]. In any case, these mice cannot allow themselves to reduce their metabolism as a consequence of the absence of UCP1: thermogenesis must be maintained. Thus, even if a component of thermogenesis that could be considered to be diet-induced thermogenesis was to be eliminated in the UCP1 KO mice, this would not result in a lower energy expenditure. In the absence of UCP1, there will still be no ‘unused energy’ to be stored, resulting in no obesity development in the UCP1 KO mice under these conditions.

The situation is principally very different with mice living at thermoneutrality. Here, any differences in thermogenesis (energy expenditure) should be directly observable since the heat is not used for thermoregulation. Thus, when mice were maintained at thermoneutrality for the experiment, fully different results were obtained. When given a high-fat diet, the UCP1 KO mice became clearly more obese than the wild-type mice (figure 2*b*) [16]—although they did not develop the massive obesity seen in *ob/ob* mice. Subsequently, several other groups obtained results fully in accordance with ours, as seen in figure 2*b–d*. Additionally, other groups [23] obtained similar results, expressed in other ways. Also a tendency to a higher body weight and lipid accumulation in UCP1 KO mice may be discerned in Zietak & Kozak [24].

## 9. Is the effect of UCP1 ablation on mouse obesity consistent?

Although the data compiled above would seem to support the concept of diet-induced thermogenesis and the obesity-promoting consequences of its absence in UCP1 KO mice, there are published data that do not accord with this. The data available on the obesity-inducing effect of UCP1 ablation have been compiled by Dieckmann *et al.* [25]. From the compilation in that paper, it is clear that the groups of L. P. Kozak and of Martin Klingenspor consistently report an absence of obesity-inducing effects of UCP1 KO, even in mice at thermoneutrality and given a high-fat diet [25–27]. These experiments were performed with different mouse strains, given different diets, etc., but in none of the cohorts was an obesity-promoting effect of UCP1 ablation seen. To our knowledge, the studies referred to here are all the published studies examining the obesity-promoting effect of UCP1 ablation. To what degree other groups have reproduced or not the obesity-promoting effect of the UCP1 KO is not known; a publication bias may exist where studies not showing effects of UCP1 ablation may not be reported.

**Figure 3.**
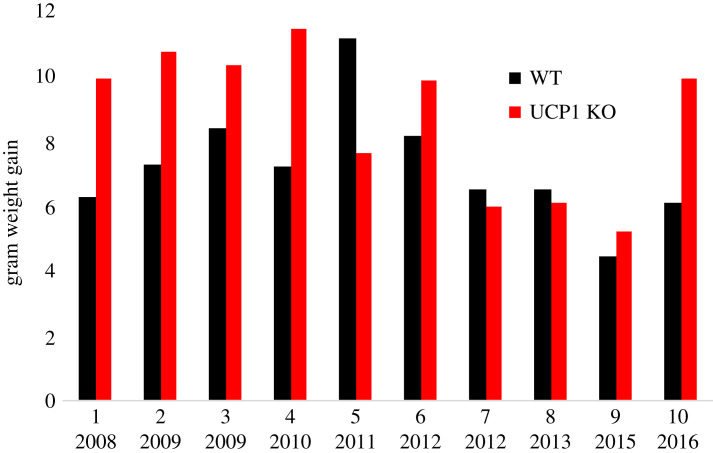
Compilation of our cohorts of wild-type versus UCP1 KO mice. Wild-type (black) and UCP1 KO mice (red) (all C57Bl/6) kept at thermoneutrality (≈30°C) were exposed on different occasions to a high-fat diet and the body weight followed for four weeks. This is a compilation of all such cohorts studied in our facility. Year stated is year of experiment. Some of these data have been included in publications: #1 and #2 [16]; #3 [28]; #4, #6 and #9 [13]; #5 unpublished—note that the wild-type mice in this cohort were not from our own breeding (for technical reasons); #7, #8 and #10 unpublished.

The contrasting data prompted us to systematically compile all our own cohorts, to examine the consistency of our results. The outcome can be seen in figure 3. As can be seen, over the years when we performed this type of experiments, 10 cohorts have been examined. All cohorts over the first 4 years yielded identical results. (An apparent exception in 2011 was a cohort where (for technical reasons) we had used a group of mice directly obtained from our supplier for the wild-type group, supposedly of the same age, etc. as our UCP1 KO mice. This was clearly not a good idea; the mice even had a higher starting weight, and we feel that we can analytically ignore this cohort but we include it here for completeness.)

However, as also seen in figure 3, around 2012 something happened. Suddenly, there was no longer an effect of the UCP1 KO on body weight. At the same time, we noted that it was not only UCP1 that was not found in these mice: the mice had become devoid of brown adipose tissue. We could no longer perform mitochondrial studies, as there was no brown adipose tissue to isolate mitochondria from. We still have no explanation to what happened. Later experiments seem to show a reappearance of the earlier situation, although a pilot test with a newly purchased batch of UCP1 KO mice from the Jackson Laboratories also failed to show an effect (not shown).

## 10. Reasons for discrepancies between outcomes

Thus, even excluding those ‘negative’ outcomes that are ‘exceptions’ because the basic criteria of a high-fat diet and thermoneutrality have clearly not been met, not all published studies show an obesity-promoting effect of the absence of UCP1. It is possible to list a long series of possible differences that may be put forward to explain why obesity is not always seen—but whether any of these are relevant cannot presently be established.

### (a) The actual UCP1 ablation

Most UCP1 KO strains that have been tested have their origin in the UCP1-ablated mouse made by Leslie Kozak [14]. This mouse was made in the classical way, with the mutation made in the 129Sv strain and then backcrossed into C57Bl/6 mice. This means that some 129Sv genes will be carried over to C57Bl/6 and they may confer properties unrelated to those related to the UCP1 KO. This was, e.g. what happened initially with the UCP2 KOs, as discussed elsewhere [29]. However, the problem with such unrelated effects should diminish with time (increased number of backcrossings) and, if anything, the 129Sv background should be protected from obesity: 129Sv mice are obesity-resistant. The Kozak UCP1 KO mouse has been distributed to many laboratories, and it is also the background for the UCP1 KO mouse obtainable commercially from The Jackson Lab. Some other UCP1 KO models are now available, e.g. from the EUCOMM programme. Such mice have been used in, e.g. the wirk of Dieckmann *et al.* [25]. Also, specially generated dual UCP1 reporter mice [30] have been used; in these strains no obesity-inducing effect has been reported to have been observed. A spontaneously occurring UCP1 KO has so far not been examined for obesity on a high-fat diet at thermoneutrality [31]. Although the method for creating the UCP1 KO should theoretically not influence the outcome, this possibility cannot fully be excluded. Concerning our own results (figure 3), some unexplained genetic drift would appear to have occurred in our colony.

### (b) The actual high-fat diet used

The obesity-proneness of UCP1 KO mice is normally tested simply by offering the mice what is generally referred to as a high-fat diet. The 45 or 60 energy% from fat Research Diets diets (D12451 and D12492) are often used; it may be noted that particularly the 45% diet is also a high-sucrose diet with a sweet taste. However, it may not only be the amount of calories from fat but also the fat source that matters. In Research Diets above diets given, the fat source is mainly lard, while in Ssniffs high-fat diet (used e.g. in [25]) where no obesity-promoting effect of UCP1 ablation is seen, it is palm oil [25–27]. In other systems, such differences in lipid source have been observed to yield quantitatively and qualitatively very different results (germ-free mice are protected against obesity on a lard diet but become obese in several other studies on a palm oil diet) [32]). Also in studies ([15,18,24] Research Diets D12331; Bio-Serv AIN-76A), a non-lard lipid source was used (hydrogenated coconut oil). Thus, the effect of UCP1 ablation may only be observed with a given type of high-fat diet—but that would, of course, make it less interesting!

### (c) The ambient temperature actually experienced

Although it is easy to state that the mice should be at thermoneutrality for an obesity-augmenting effect of the UCP1 ablation to become manifest, the practical implication of this is not easy. The actual thermoneutral zone of mice is not firmly established but is normally stated to be around 30°C (e.g. [19]). A temperature of 27°C may thus perhaps be considered somewhat too low; we have therefore excluded experiments performed at this temperature from the present discussion. This scientific uncertainty of the actual borders of the thermoneutral zone may for several reasons not be so surprising. Temperature has the benefit that it is tantalizingly easy to measure and state with even decimal accuracy—but that makes it easy to forget that what the mice experience is a multifactored microclimate. Particularly, the introduction of individually ventilated cages (IVC) with often very high ventilation rates means that a wind-chill factor may be introduced in the cages. Studies clearly indicate that mice in their home cages in rooms at normal housing temperature physiologically experience a colder temperature than they did with traditional housing [33]. To what degree this also occurs when the housing temperature is nominally 30°C has not been experimentally investigated but, as discussed above, even minor cold stress may make diet-induced thermogenesis effects invisible.

### (d) The duration of the experiment and the age of the mice

**Figure 4.**
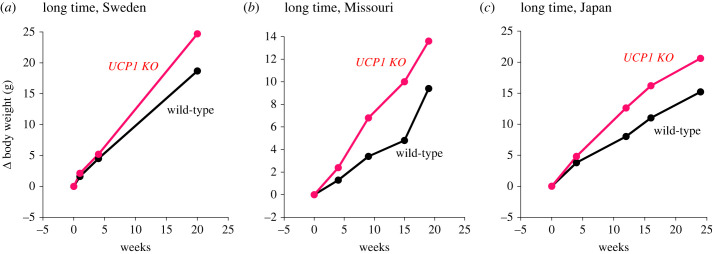
Late manifestation of the obesity-inducing effect of UCP1 ablation. High-fat-fed wild-type and UCP1 KO mice (all C57Bl/6) were followed principally as those in figure 2 but studied for longer periods. (*a*) Mice studied at thermoneutrality in our laboratory. Our unpublished observation, 2016. (*b*) Mice (female) studied at 25°C. Redrawn from Winn *et al.* [34]. (*c*) Mice studied at 23°C. Redrawn from Kontani *et al.* [35].

The standard experimental time used by us has been approximately four weeks, normally starting with ‘young adult’ mice at an age of 12 weeks. Evidently the age of the mice may affect the outcome. For example, we have found that a cohort that initially did not show marked effects of the UCP1 ablation did display robustly higher body weights in the UCP1 KO mice after some further 15 weeks (figure 4*a*). Two other observations of late manifestation of obesity (figure 4*b,c*) are remarkably interesting in that these mice were not kept at thermoneutrality but at 25°C and 23°C. How prolonged time/high age can unmask a potential obesity-inducing effect of UCP1 ablation even in mice living below thermoneutrality is not presently understood.

### (e) The microbiota (or similar local effects)

Even if all conditions that can be controlled are set to the expected values, ‘unknown’ factors may influence the outcome of metabolic experiments. Regarding other parameters than those studied here, it has been demonstrated that even the same strain of mouse from the same supplier when examined in different laboratories can display different outcomes [36]. This is presently ascribed to local microbiota effects but other ‘unknown’ factors cannot be excluded.

### (f) The prehistory of the mice

Similarly, what the mice have earlier experienced may make them prone to different outcomes. For instance, the mice may be the offspring of heterozygous matings. This setting is often considered to be the most genetically secure but it should be remembered that the UCP1 KO mouse pups may be in a weaker state than their wild-type siblings and this may affect their later response to feeding. Indeed, the UCP1 KO mice used by Dieckmann *et al*. [25] were significantly smaller at weaning than their siblings. The alternative, homozygous lines of wild-type and UCP1 KOs may instead lead to a higher risk for genetic drift—although this should be minimized by frequent backcrossing.

### (g) The strain of mice

See below in discussion of the adipostat.

However, given all of the above issues, it must clearly be stated that if the obesity-inducing effects of UCP1 KO are so elusive that they are only manifest under very specific conditions, it is difficult to ascribe major biological significance to the phenomenon, even in a mouse system.

## 11. Opposing powers: UCP1 versus the adipostat

As stated above, it seems evident that in the absence of a fraction of the food being combusted in the brown adipose tissue, the mice would inevitably become obese. However, this prediction ignores the other regulatory systems of the organism. One of these is the adipostat (lipostat) system: the system that apparently senses the total amount of lipid reserves in the body and adjusts food intake thereafter. It may be said that it is the apparent malfunction of this adipostat function in male C57Bl/6J (black 6) mice that makes them the preferred strain for the study of obesity: they allow themselves to become fat. Thus, it is not surprising that the effect of UCP1 KO is most striking in C57Bl/6 mice—but here again: if the use of this strain is a necessity to observe the effects, are they then biologically important? (But, of course, this is a comment that can be forwarded concerning very many of today's ‘preclinical’ obesity experiments that nearly all use C57Bl/6 mice.)

So, what happens in more obesity-resistant mouse strains? The expected outcome would then be that these UCP1 KO mice would be better in maintaining their body weight and would do this by having a lower food intake. The only published study, performed in the obesity-resistant 129Sv mice, supports this prediction [37], but we have also seen that in the FVB/N strain, the absence of UCP1 leads to a decrease in food intake of a cafeteria diet such that the mice in this way become protected against obesity (our unpublished observation, 2009).

## 12. If obesity is associated with *recruited* brown adipose tissue, why is atrophied brown adipose tissue often reported in obese animals/man?

The tenet described here in general is that obesity normally leads to increases in brown adipose tissue recruitment (UCP1 amount), partially counteracting the obesity. Correspondingly, certain obesities could thus be explainable as being due to a (partial) lack of UCP1 amount/activity.

Such suggestions were initially apparently well supported by observations with genetically obese mice, the *ob/ob* mice. Both physiological and biochemical data initially indicated that the explanation for the obesity in these mice should be the absence of a thermogenic effect of leptin that in its turn should have occurred through activation of brown adipose tissue. However, further analysis of the available data leads to the conclusion that leptin is not thermogenic and that the obesity in the *ob/ob* mice cannot be understood as being due to brown adipose tissue inactivity [38–40]. (It should be added that all these experiments were performed on the inbred C57Bl/6 strain; concerning the *ob/ob* gene on the outbred Aston mice strain, lowered brown adipose tissue thermogenic activity may contribute to the obesity (see summary in [41]).) A similar series of observations has been made concerning the obesity induced in glucocorticoid-treated mice, where earlier studies implied an atrophy of brown adipose tissue that could (partly) explain the obesity. However, further studies have again indicated that the brown adipose tissue is not atrophied—it only appears to be so, due to lipid accumulation [42,43]. Thus, the conclusions concerning both the *ob/ob* mouse and the glucocorticoid-treated mouse are that the brown adipose tissue is not actually atrophied and absence of brown-fat-derived thermogenesis can thus not explain the observed obesity. We refer to these states as states of pseudo-atrophy.

*Does diet-induced ‘whitening’ exist?* A phenomenon termed ‘whitening’ is often referred to. This is used to describe the alterations in brown adipose tissue that occur when mice are exposed to a high-fat diet. The tenet is that a high-fat diet would lead to alterations that would decrease the thermogenic capacity of the tissue. This would thus constitute a self-reinforcing process where high-fat diet ingestion would reduce diet-induced thermogenesis and thus promote even more fattening. However, it is far from certain that this phenomenon exists. The tissue in mice with diet-induced obesity does indeed *look* atrophied, with large fat droplets, less cytosol per visible area in a microscopy slide, less UCP1 staining per area, less vascularization and innervation per area. However, all these effects can be understood as being secondary to the lipid accumulation in the tissue and the ‘dilution’ of all other tissue constituents that this leads to. There may also be decreased UCP1 gene expression (per unit RNA) and decreased UCP1 protein amounts (per unit protein). However, such observations are also understandable as ‘dilution’ effects, due to e.g. enhanced expression of genes for fatty acid synthesis. Thus, if these results are recalculated on a whole-tissue (depot) basis, there may not be any lowering of UCP1 amounts at all (but such calculations are not generally made). The mice with whitened brown adipose tissue are also reported to present with lower metabolism (energy expenditure) [44,45], but this is generally expressed—physiologically erroneously—per body weight, a calculation process that automatically makes obese mice demonstrate low metabolic rate (as has been discussed elsewhere, e.g. [46–49]). All these observations can be summarized to imply a decreased capacity for (adrenergically induced) non-shivering/diet-induced thermogenesis, but to our knowledge this has not been experimentally verified. Instead, the general picture for the high-fat diet-induced whitening appears to be very similar to that observed in the *ob/ob* mice and glucocorticoid-treated mice discussed above, i.e. the ‘whitened’ mice are pseudo-atrophied. Similarly, there is no reason to assume that whitening in obese mice may be caused by increased thermal insulation of the mouse due to the extra lipid, since there is no demonstration that obesity insulates; on the contrary, it has been demonstrated that obesity does *not* insulate, at least not in mice (and probably not in humans either) [50,51].

## 13. Conclusion concerning mice

From the data collected above, there is reason to assume that the phenomenon of diet-induced thermogenesis can be observed in mice under certain conditions, that it then is mediated via UCP1, and that a genetic reduction of UCP1 may promote obesity in mice. Despite this, there are no confirmed pathological conditions in mice where obesity is caused by a lower activity of the brown adipose tissue thermogenic system (as compared to its total absence in the UCP1 KO mice). Although the absence of such reported effects would seem to make the entire concept of diet-induced thermogenesis less biologically interesting, it should be remembered that nearly all metabolic experiments in mice have been and still are performed at temperatures below thermoneutrality, where any effects of changes in diet-induced thermogenesis are principally invisible. Thus, a true obesity-inducing effect of (unknown) alterations in UCP1 amount or its inherent or endogenously stimulated activity has not as yet been identified.

## 14. Human obesity and brown adipose tissue

The above studies in mice may be interpreted that, at least under certain conditions, diet-induced thermogenesis exists and is UCP1-mediated, and that in the absence of this process, mice may become more prone to obesity.

Correspondingly, already from early compilations of the relationship between the presence of brown adipose tissue in different subjects and their degree of obesity [52], it was suggested that a correlation exists: that obese people have less brown adipose tissue, with the implication that the low amount of brown adipose tissue (partly) could explain the obesity. However, it was also seen that both the amount of brown adipose tissue, as well as the degree of obesity, correlate with age: less brown adipose tissue with age, more obesity with age [52]. The correlations may thus be just correlations and no causality may exist.

These early correlations were based on histological observations of UCP1 in neck cervical adipose tissue depots. Many more studies have later been done based on data from fluorodeoxyglucose positron emission tomography (FDG PET) scans, i.e. by following uptake of glucose into tissues. Such studies can either be retrospective or dedicated [53], and the outcomes may be affected by these differences in study layout.

*Problems with retrospective PET scan studies.* In the retrospective studies, archived hospital scans are reanalysed for indications of brown adipose tissue glucose uptake, as a sign of thermogenic activity. The outcome is then correlated with metabolic data (i.e. mainly BMI) from the patients (the PET scans are generally mainly obtained from patients monitored for cancer metastases, in itself of course giving a bias). The main problem with this type of data is that they represent only the acute thermogenic activity of the patient. This means that if the patient ‘feels’ warm during the examination, brown adipose tissue, even if it is present, will not be visible, as it is not stimulated. Firstly, this means that only a small fraction of the true brown-fat-positive patients will be identified. Secondly, of particular relevance for the discussion here, the analysis of the outcome (correlation with obesity) may be systematically affected by the obesity itself. In contrast to what is normally assumed, obese persons normally have a higher basal metabolic rate than lean people (e.g. [54]). Given that the obese and the lean are exposed to the same environmental temperature when examined by PET scans, the lean may activate brown-fat thermogenesis, whereas the obese may not need to do this, due to the already higher metabolic rate. The visibility of brown adipose tissue will therefore depend on the obesity, and this will promote the negative correlation between brown adipose tissue and obesity—but with the inverse causation: the obesity reduces the brown-fat signal. As indicated above, it may be thought that the obesity also leads to higher thermal insulation and through this may quench the signal—but in our opinion, obesity does not thermally insulate humans [51].

In dedicated studies, the amount of brown adipose tissue is determined in subjects exposed to so much cold that their brown adipose tissue is definitely activated (if they have any). This thus eliminates the problems described above for the retrospective studies. However, what will be measured is the amount of brown adipose tissue that can be activated by cold, and this is not necessarily what is relevant in the present context. Indeed, based on implications from mouse experiments [28], humans could have significant amounts of cold-induced brown adipose tissue thermogenic capacity but may be incapable of activating it for diet-induced thermogenesis. Data presented by Loeliger *et al*. [12] would indicate this. Thus, estimates of brown adipose tissue thermogenic capacity obtained during cold stimulation may be irrelevant for examining the significance of brown adipose tissue for diet-induced thermogenesis in humans.

*Does brown-fat-mediated diet-induced thermogenesis exist in humans?* Based on the criteria discussed above for facultative diet-induced thermogenesis in mice, the human version should also be both facultative and adaptive, and it should be identifiable as an extra component on top of the obligatory thermogenesis. Most studies examining diet-induced thermogenesis in humans have not had possibilities to distinguish between the obligatory and facultative components (e.g. [55]). However, that a facultative adrenergic component may exist has classically been implied [56], and this could thus be mediated by acute brown adipose tissue activation. Only a few studies have approached the issue of feeding-induced brown adipose tissue activation, and these studies have mainly used FDG PET scans to follow brown adipose tissue activity. However, the radioactive glucose will, particularly after a meal, also be taken up into the muscles, and this disturbs the interpretation [57]. This complication is avoided if instead radioactive oxygen is followed, and in studies using this substance, clear indications of meal-induced activation of human brown adipose tissue have been seen [58]. Thus, a facultative component of human diet-induced thermogenesis does seem to exist.

*How does human brown adipose tissue thermogenic capacity relate to obesity?* Based on what has been demonstrated in mice, it would further be expected that healthy humans should show a *positive* correlation between obesity and brown adipose tissue thermogenic amount (i.e. that the thermogenic capacity would be adaptive). We are only aware of one study that has arrived at such a conclusion. Sanchez-Delgado *et al*. [59] observed a positive correlation between percentage fat mass in humans and brown adipose tissue volume; it may, however, be discussed whether the volume of the brown adipose tissue is the most relevant parameter for estimating brown adipose tissue thermogenic capacity since the volume is determined primarily by the accumulated lipid.

An additional and only recently formulated complication is the negative interaction between obesity and glucose uptake. Glucose uptake induced by norepinephrine in brown adipose tissue is not, as was initially expected, due to a UCP1-dependent combustion of glucose in the tissue. Rather, glucose uptake occurs even in the absence of UCP1 and thus in the absence of thermogenesis [60,61]. There is good reason to think that the glucose uptake observed during PET scans is mediated to a large extent by insulin stimulation (e.g. discussion in [62]). This means that insulin resistance caused by the obesity may lead to lower glucose uptake into brown adipose tissue, and this will thus create a false negative correlation between brown adipose tissue activity and obesity.

Thus, merely to observe a correlation between obesity and brown adipose tissue in humans, we have to have adequate measures of both parameters. Obesity as such is not difficult to measure, but, as can be understood from the above, a relevant estimation of brown adipose tissue in humans is still quite a difficult issue. And as even correlations are thus difficult to obtain, an answer to the question of causation is even more remote.

## 15. Contestable genetic evidence for UCP1 involvement in human obesity

Given the problems with the direct estimation of brown adipose tissue significance, genetic methods may be relevant. Firstly, it may be noted that the UCP1 gene itself has never appeared as a candidate gene in any genome-wide association study (GWAS) for obesity-related issues. To date, the only GWAS UCP1 association found has been to some measures of physical activity where a single nucleotide polymorphism has been found close to the UCP1 gene [63]; these observations have not been independently confirmed.

There has instead been quite some interest concerning a polymorphism in the upstream region of the UCP1 gene: the A3826G polymorphism. Evidence has been published that the A allele of this polymorphism is associated with higher levels of expression of UCP1 [64]. Accordingly, young Japanese females who carry the A allele have been reported to both eat more and to have a higher energy expenditure [65], and young Japanese boys with the A allele display a higher postprandial increase in energy expenditure after a high-fat meal [66]. It has also been reported that adult subjects carrying the A allele had a somewhat higher chance of being in the population quartile with the lowest BMI [67]. All of this would in itself be a good indication of the significance of UCP1 and brown adipose tissue for human metabolism and obesity propensity. However, compilations of all available data (some 60 papers) concerning the A3826G polymorphism and its possible metabolic roles seems to indicate that there is no consistent effect of this polymorphism on human obesity ([68] and our unpublished analysis, 2023), and that the reported correlations may mainly be statistical coincidences.

Concerning the coding region of UCP1, no human UCP1 KO has so far been reported. A series of mutations in the coding region of the human UCP1 gene have been described but the metabolic outcome of these has until now not been convincingly established [68].

## 16. The real question: do UCP1 and brown adipose tissue activity protect against obesity in humans?

Based on the summary here of the available data, it may be concluded that at least in mice, diet-induced thermogenesis may be observable under certain conditions and is as such located to brown adipose tissue, and its absence (as in UCP1 KO mice) may lead to obesity. In humans, correlations between obesity and (low) brown adipose tissue activity have been published, but causation has not been demonstrated. However, meal-induced brown-fat-derived thermogenesis does seem to occur in humans, and its presence and absence could therefore—everything else being equal—affect the propensity to obesity in humans. The total thermogenesis from brown adipose tissue in adult man has been estimated to correspond to a change in body weight of somewhere from about 1 (most estimates) up to 10 kg per year. Thus, brown adipose tissue may, even in humans, to some extent protect us against obesity, and a reduced brown adipose tissue activity may—again everything else being equal—aggravate the development of obesity in humans.

## Data Availability

This article has no additional data.
